# Color matching of bicomponent (PET/PTT) filaments with high performances using genetic algorithm

**DOI:** 10.1038/s41598-024-61608-z

**Published:** 2024-05-13

**Authors:** Marwa Souissi, Sabrine Chaouch, Ali Moussa

**Affiliations:** 1 Laboratory of Environmental Chemistry and Clean Processes, University of Monastir, Monastir, Tunisia; 2 National Engineering School of Monastir, University of Monastir, Monastir, Tunisia; 3 Textile Engineering Laboratory, University of Monastir, Monastir, Tunisia

**Keywords:** Bicomponent polyester filaments, Dyeing, Genetic algorithm, Color matching, Color recipe prediction, Chemistry, Engineering

## Abstract

In recent years, a great interest has focused on the use of bicomponent filaments in several high-performance textile articles such as swimwear, sportswear and even high-quality denim. To dye fabrics containing these filaments, it is necessary to establish appropriate dye recipes allowing to obtain desired shades. In this article, we developed a genetic algorithm to optimize the color matching step of these bicomponent filaments, especially (PET/PTT) filaments. Three disperse dyes with different molecular weights were used for dyeing. The objective is to reproduce the reference color by choosing the appropriate disperse dyes among the available dyestuffs and their corresponding quantities to use on the mixture. For modeling, two sets of parameters (lied to the color formulation problem and the genetic algorithm), the objective function as well as the different stages of the algorithm were defined and described. In addition, different techniques of selection and mutation were applied and evaluated. The optimization criterion is to reduce the CMC color difference between the desired reference colors and the colors proposed by the algorithm. The developed algorithm showed good performances with very small color differences. The results indicate that the roulette wheel selection technique outperforms both rank and uniform selection methods. Moreover, employing a simple mutation strategy yields favorable outcomes with CMC color differences all lower than 1.

## Introduction

In recent years, conventional polyester filaments would no longer be able to satisfy the textile market demand, which is constantly evolving and changing. To meet this demand, so-called bicomponent, tricomponent and even more filaments, combining several types of polyester have been developed. These filaments present excellent performances in terms of durability^1^, mechanical resistance^2,3^, water vapor permeability^4^, elastic recovery^5,6^, biodegradability^7^, thermal comfort^6^, etc. In this context, a new trend is emerging; it consists in using these functional filaments which have new properties for technical use. This research work focuses on bicomponent filaments composed of two polymers: polyethylene terephthalate (PET) and polytrimethylene terephthalate (PTT). These two filaments are adjacent, arranged side by side and extruded from the same spinneret^5^. The resulting bicomponent filaments (PET/PTT) exhibit a spiral morphological structure which gives them significant elastic properties^6^ and allows to bring the stretch effect to the various articles while guaranteeing a better behavior during the various treatments^7^.

Among existent studies, several works have focused on the characterization and the physico-chemical properties of these bicomponent filaments (PET/PTT)^8–11^. Indeed, recent studies investigated the effect of spinning parameters on the final structural of bicomponent filaments (PET/PTT) and the interfacial structure and binding forces between polytrimethylene terephthalate (PTT) and polyethylene terephthalate (PET) filaments using sophisticated analytical techniques^9,10^. Obtained results showed that during the spinning process, a distinct interface layer formed between PTT and PET, crucial for their binding. Sufficient blending time led to an ester-interchange reaction, yielding copolymers. Other innovative study^11^ has established a rapid identification of bicomponent fibers (PET/PTT) types and their spatial distribution structures. Subsequently, an artificial intelligence software computed the mass percentage of each component based on density, cross-sectional area, and total number of test samples for each component.

In previous studies conducted by the authors and their collaborators^12^, a comprehensive characterization of these filaments was conducted, encompassing mechanical, physical, thermal, and chemical analyses. The results obtained highlighted the considerable potential of these bicomponent filaments, showcasing superior elasticity and elastic recovery, along with a crystallinity rate and glass transition temperature lower than those observed in other polyester filaments. This research team has even investigated^13,14^ the development, modeling and optimization of an economical and clean process for dyeing bicomponent filaments at a temperature equal to 100 °C. Obtained results showed that *p-vanillin* is an excellent alternative to remedy the use of toxic carriers and to maintain the physico-chemical characteristics of the bicomponent filaments.

According to all these research works carried out recently concerning bicomponent filaments, it is obvious that these latter present an excellent alternative for replacing elastane filaments which are causing more and more problems in dyeing and in textile finishing. It is also evident the great need to solve all the problems related to the textile finishing of these filaments increasingly used in high performance and excellent quality articles. In this context, no studies on the color formulation of bicomponent filaments are available in the literature.

This paper presents an original work whose objective is to apply artificial intelligence technique in order to predict the appropriate color recipes for dyeing bicomponent filaments (PET/PTT) with a mixture of disperse dyes allowing to obtain desired shades. So, a genetic algorithm was developed to solve this color matching problem. The goal is to optimize the step of color matching by predicting the best dyeing recipe in multicomponent mixtures. The proposed algorithm should determine which dyes to use from the range of available dyes and their corresponding concentrations. In this study, three disperse dyes were used: CI Disperse Red 167.1, CI Disperse Yellow 211 and CI Disperse Blue 79.1. The efficiency of the developed genetic algorithm was evaluated and proved using the CMC color difference between the desired reference colors and the predicted recipes.

## Materials and methods

### Textile support

Based on 100% bicomponent (60% PET, 40% PTT) filaments, jersey knits are made with circular knitting machine type Tricolab gauge 12 (Sodemat, France). The knits are used for dyeing in order to evaluate the performances of the developed algorithm. These multifilament yarns had a linear density of 83 dtex and contained 64 filaments per yarn. The knit thickness (mm) and the knit weight (g/m^2^) are equal to 0.92 and 215, respectively. Table 1 summarizes the principal thermal, mechanical and physical characteristics of these textile supports that have been realized during subsequent work^12^.

**Table 1:** Principal characteristics of bicomponent (PET/PTT) filaments^12^.

Analysis	Parameters	Values
Thermal characteristics	Melt temperature (°C)	222 (PTT) 248 (PET)
	Glass transition temperature (°C)	37 (PTT) 62 (PET)
	Melt enthalpy (J/g)	55.32 (PTT) 55.27 (PET)
	Cristallinity (%)	37.78 (PTT) 47.27 (PET)
Mechanical characteristics	Tenacity (CN/tex)	30.80 ± 0.25
	Elongation at break (%)	44.52 ± 0.34
	Recovery elasticity after 1 cycle	69.23 ± 0.09
	Recovery elasticity after 10 cycles	58.42 ± 0.15
	Permanent deformation (%)	2.16 ± 0.15
Physical characteristics	*2θ*_*1*_ (°)	17
	*2θ*_*2*_ (°)	23.4
	*2θ*_*3*_ (°)	25.2
	Cristallinity (%)	57
	*a* (Å)	4.84
	*b* (Å)	6.08
	*c* (Å)	13.40

With *2θ*_*1*_, *2θ*_*2*_ and *2θ*_*3*_ are the angles of the diffraction peaks and *a, b* and *c* are the parameters of the crystal lattice.

### Used dyes

**Figure 1:**
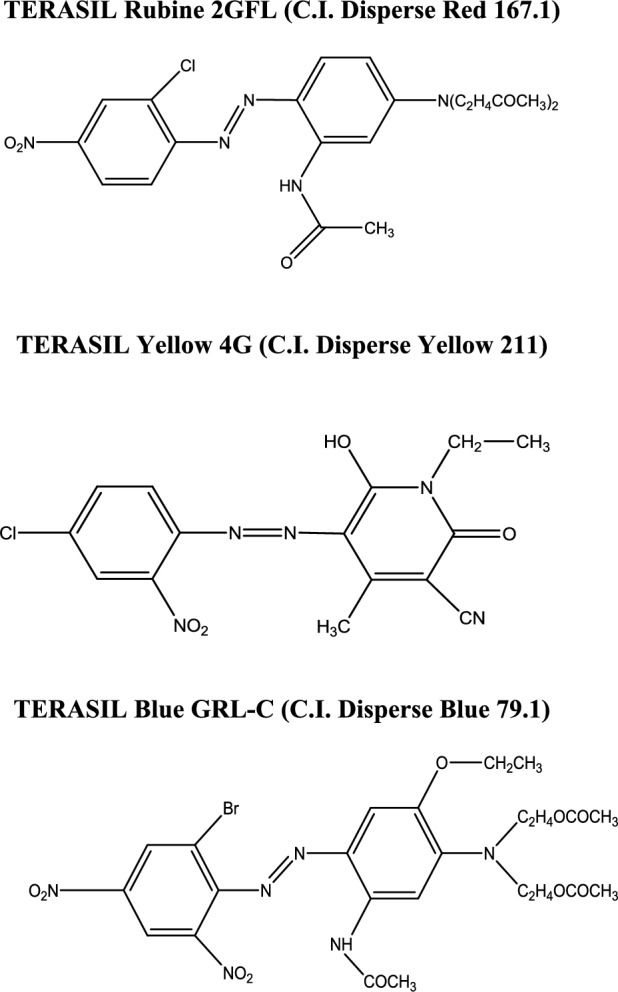
Chemical structures of disperse dyes.

The disperse dyes used throughout this study are of the Terasil type manufactured by the Huntsman Company (Germany). These colorants are: Terasil Rubine 2GFL (C.I. Disperse Red 167.1), Terasil Yellow 4G (C.I. Disperse Yellow 211) and Terasil Blue GRL-C (C.I. Disperse Blue 79.1). These dyes exhibit vivid colors with deep shades. The chemical structures of used dyes are presented in Fig. 1.

### Dyeing procedure

**Figure 2:**
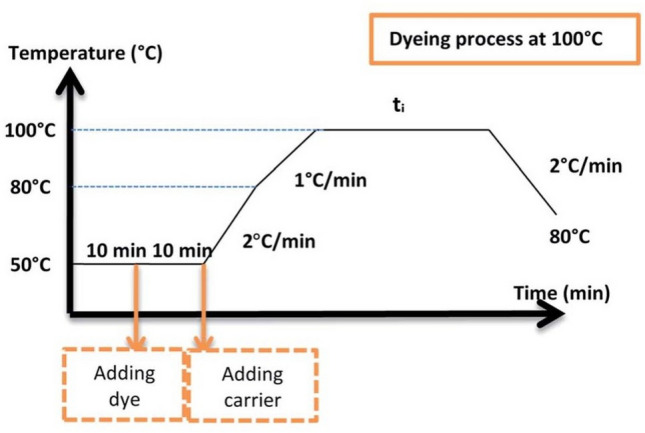
Dyeing process of bicomponent filaments with used disperse dyes.

**Figure 3:**
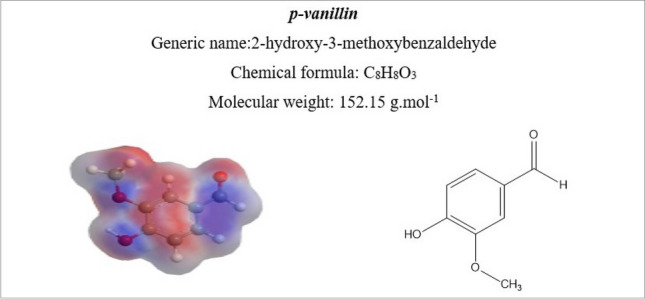
Chemical formula of *p-vanillin*.

Bicomponent polyester filament knits (PET/PTT) were employed for dyeing with disperse dyes across various shades, ranging from 0.05% to 3%. This process facilitated the creation of a database, which will subsequently serve as the research foundation for our proposed genetic algorithm. The algorithm aims to reproduce different reference colors with minimal color differences. Dyeings were carried out using an Ahiba dyeing machine (Datacolor, USA). For color reproducibility, each dyeing was realized thrice. The dyeing process used is illustrated in Fig. 2. It is an ecological process already established in previous research works^15,16^. In this process, conventional toxic carriers have been replaced by biosourced one, namely *p-vanillin*, to improve the dyeing performances of bicomponent polyester filaments. Figure 3 shows the chemical formula of *p-vanillin.* After dyeing, the samples were post-treated at 50 °C for 10 min using 2 g L^−1^ sodium hydrosulphite and 2 mL L^−1^ sodium hydroxide solution (36°Be) with a liquor-to-fiber ratio of 20:1.

### Color measurement

For each dyed sample, the color strength (*K*/*S*) allows to evaluate the dyeing depth of the dye fixed on the fiber. This parameter can be determined by measuring the reflectance *R* of the dyed sample at a well-determined wavelength. The relation linking the color strength (*K*/*S*) to the reflectance *R*, known as the Kubelka–Munk formula, is presented by the following formula^17^:

1 $$\frac{K}{S}=\frac{{\left(1-R\right)}^{2}}{2R}-\frac{{\left(1-{R}_{0}\right)}^{2}}{2{R}_{0}}$$

where *K*, *S* and *R* are respectively the absorption coefficient, the scattering coefficient and the reflectance of the dyed sample, and *R*_*0*_ is the reflectance of the sample before dyeing.

All colorimetric measurements were performed using a spectrophotometer type Spectraflash 600 Plus (Datacolor International, USA) with D65 light at a 10° observer, using d:8° geometry, specular component included (SCI), ranging from 400 to 700 nm at 10 nm intervals. Each measurement was repeated thrice. Values presented throughout this study are the average of three obtained values of the coloring strength. To evaluate the color difference between the desired shade and the reproduced color, the Δ*E*_*CMC(2:1)*_ formula was used^17^.

## Development of the genetic algorithm for color formulation problem

### Color formulation problem

In the textile industry, color is often one of the most textile design fundamental aspects which contributes enormously to the finished fabric overall visual effect. Color formulation step corresponds to find dyeing recipes which allow to get the color as close as possible to the desired reference color. Indeed, it is an optimization problem consisting in the combinatorial selection of components (dyes) and their quantities (concentrations) to obtain an exact color match between the resulting color and the reference one.

Currently, there are various methods for color matching problem: conventional methods such as spectrophotometric or colorimetric methods^18–21^ and artificial intelligence (AI) methods^22–25^.

In this paper, a genetic algorithm was developed and applied to resolve the color formulation problem in the case of bicomponent (PET/PTT) filaments. The aim is to select the disperse dyes to be used in mixture and their corresponding concentrations to optimize the color formulation step of bicomponent filaments.

Before applying the proposed algorithm, the different parameters and the criterion of optimization must be fixed. As the aim of this study is to optimize and to predict the optimal dyeing recipe i.e. the appropriate adding quantities of dyes available for dyeing. The best recipe is the one that minimizes the *ΔE*_*CMC(2:1)*_ value between the reference and the reproduced color. These parameters are presented in Table 2.

**Table 2:** Parameters of the color formulation model.

Symbols	Descriptions
*K*	Number of dyes available for using in mixture;
*D*_*j*_	Dyestuff index *j*, *j* ∈ {1, …, *K*};
*n*	Number of concentrations available for each dyestuff;
*C*_*ij*_	Concentration index i of dyestuff *D*_*j*_, *i* ∈ {1, …, *n*}, *j* ∈ {1, …, *K*};
Δ*E*_*CMC*(2:1)_	The CMC(2:1) color difference between the reference and the reproduced color. The objective of our algorithm was to propose the best color recipe allowing minimization of this Δ*E*_*CMC*(2:1)_ value

### Description of the applied genetic algorithm

The genetic algorithm is an optimization algorithm inspired by the evolution process of living beings^26^. It is an adaptive heuristic search based on the mechanisms of natural selection and genetics^27^. It combines a strategy of "survival of the fittest" with a random and structured information exchange. For a problem where a solution is unknown, a set of possible solutions is randomly created. This set is called the population. The characteristics (or variables) are then used in gene sequences which will be combined with other genes to form chromosomes and subsequently individuals. Each solution is associated with an individual, and this individual is evaluated and ranked according to its resemblance to the best, but still unknown, solution to the problem. After a certain number of iterations, a generation with a more suitable individuals will be proposed^28^. The use of a natural selection process allows to converge gradually to the best solution^29^.

To resolve the color formulation problem by applying the GA, it is assumed that each individual is formed by a single chromosome corresponding to a color recipe; the number of genes in chromosome is equal to the number of available dyestuffs.

Adding to parameters already previously presented in Table 2, a second set of parameters lied to the GA are fixed. These latter are presented in Table 3. *N* and *T* were optimized using an experimental design method. Their variation levels are presented in Table 4. The optimization of the parameters values was realized using MINITAB software.

**Table 3:** Parameters of the genetic algorithm.

Symbols	Descriptions
*T*	Total number of generations (iterations) in the genetic algorithm;
*P*_0_	Initial population;
*P*_*t*_	Population of the generation index *t*, *t* ∈ {1,…,*T*};
*N*	Total number of individuals in the population;
*x*_*i*_	Individual index *i*, *i* ∈ {1,…,*N*};
*n*_*c*_	Total number of chromosomes;
*c*_*j*_	Chromosome index *j*, *j* ∈ {1,…,*n*_*c*_};
*n*_*g*_	Total number of genes;
*g*_*k*_	Gene index *k*, *k* ∈ {1,…,*n*_*g*_}

**Table 4:** Genetic algorithm parameters and levels used in the full factorial design study.

Factors	Symbol	Number of levels	Variation levels				
			1	2	3	4	5
Total number of individuals	*N*	5	50	100	200	500	1000
Total number of generations	*T*	5	5	10	20	50	100

**Figure 4:**
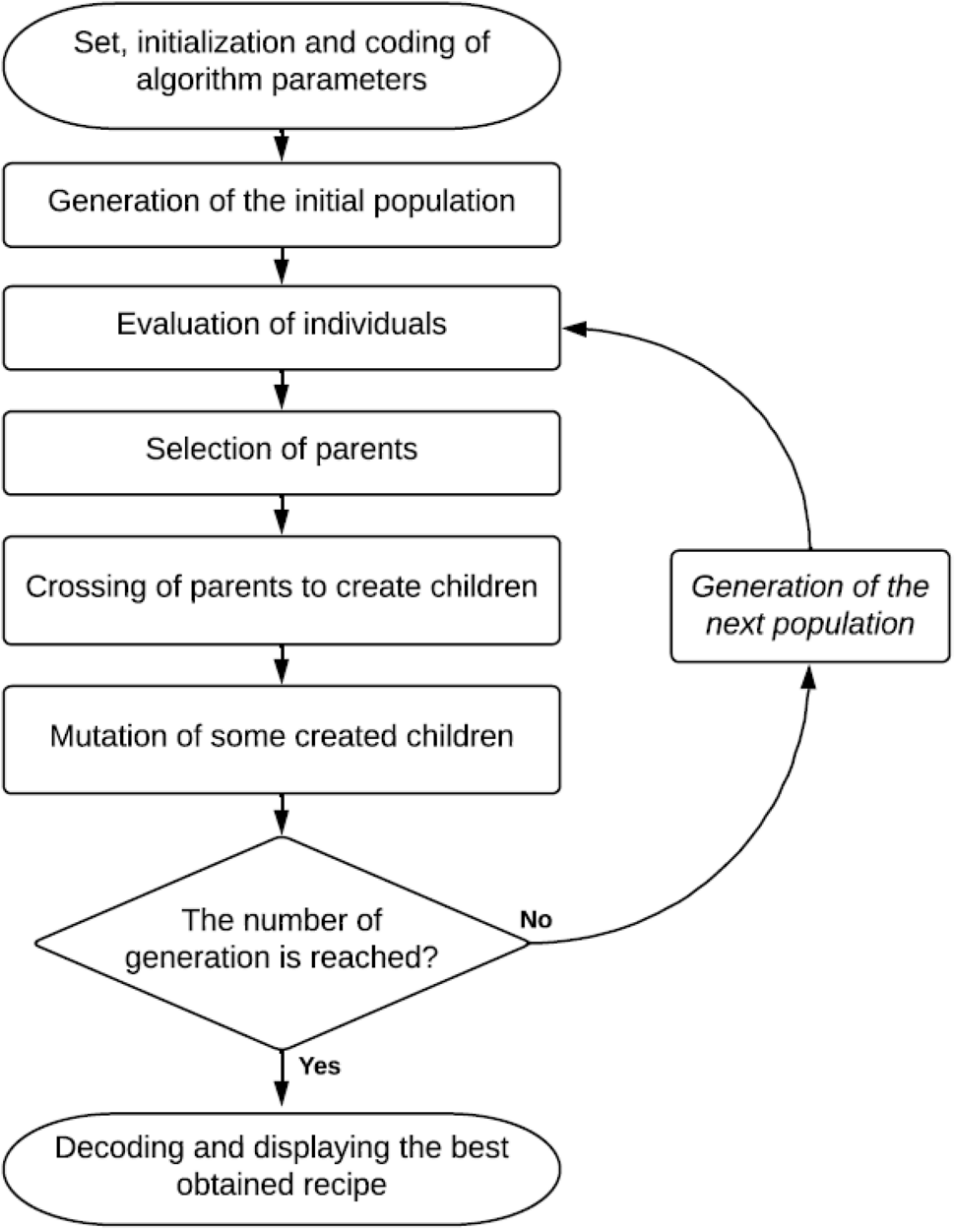
Flow chart of the genetic algorithm.

The proposed genetic algorithm, illustrated in Fig. 4, can be described as follow:

- *Stage 1*: Set, initialization and coding of GA parameters
  At first, *T* (number of generations) and *N* (number of individuals in the population) are initialized and the genetic algorithm parameters are coded. In this study, each individual was coded using one chromosome. This latter contains a certain number of genes; each gene defines the concentration of each dyestuff used in mixture. Values of concentrations vary from 0 to 3%. For coding these concentrations, we opted for a list of integers; each used allele was from 1 (i.e. concentration of 0%) to the total number of possible concentrations (i.e. concentration of 3%).
- *Stage 2*: Generation of the starting population
  The starting population *P*_*0*_, composed of *N* individuals, was generated randomly, covering the entire range of possible solutions. In fact, a random color recipe was assigned for each individual of the population *P*_*0*_.
- *Stage 3*: Evaluation of individuals
  The evaluation of individuals was made by calculating for each individual *x*_*i*_ the function *F(x*_*i*_*)* as follows:
  2 $$F\left({x}_{i}\right)= \frac{1}{\Delta {E}_{CMC\left(2:1\right)}+\varepsilon }$$
  where $\Delta {E}_{CMC\left(2:1\right)}$ is the color difference between the standard color and the dyeing recipe proposed by the individual *x*_*i*_, and *ε* is a negligible positive value.
  Then, the quality of the solution proposed by *x*_*i*_ is proportional to the value of *F(x*_*i*_*)*.
- *Stage 4*: Selection of parents
  The selection process determines the set of chromosomes from the actual population for reproduction. So, by using a selection method, *r* different couples of parents are selected to form a reproduction set *R*. There are several selection techniques^30^: fitness proportionate selection (roulette wheel selection, stochastic universal sampling), rank selection, tournament selection, uniform selection, etc.
  In order to choose the best selection method for our case, a comparison between different selection techniques was made as shown in Table 5.

**Table 5:** Study of different genetic algorithm operators.

GA operators	Studied techniques
Selection	Roulette selection technique
	Rank selection technique
	Uniform technique
Mutation	Without mutation
	Simple mutation
  - Roulette wheel selection of parents
    In this selection technique, the selection of parents from the actual population was made randomly to generate the new population based on the probabilities *p*_*1*_*(x*_*i*_*)* as follows:
    3 $${p}_{1}\left({x}_{i}\right)= \frac{F({x}_{i})}{\sum_{k=1}^{N}F({x}_{k})}$$
    where *F(x*_*i*_*)* is the performance function value of the individual *x*_*i*_ (*i* ∈ {1,…,*N*}).
  - Rank-based selection of parents
    The selection of parents from the actual population was made randomly to generate the new population based on the probabilities *p*_*2*_*(x*_*i*_*)* as follows:
    4 $${p}_{2}\left({x}_{i}\right)= \frac{2 (N-k+1)}{N(N+1)}$$
    where *k* is the rank of the individual *x*_*i*_ (*i* ∈ {1,…,*N*}) based on its performance function value *F(x*_*i*_*)*.
  - Uniform selection of parents
    It is a random selection, uniform and without intervention of the adaptation value (the evaluation function *F(x*_*i*_*)*); each individual has the same probability of being chosen.

- *Stage 5*: Crossing of parents to create children
  After the selection of parents, a cross over operator was applied for each couple. It consists in combining two chromosomes in order to generate a new off spring. The aim is to produce a new chromosome more efficient than chromosomes of parents by selecting the best characteristics of each of them^31^. Among the existing crossing methods^32^, we applied in this study a single cross over point chosen randomly. In fact, to reproduce a child *e* based on a couple of parents (*p1*, *p2*), we started by choosing a random number which will correspond to the crossover point. The child *e* thus created will be composed of a sequence of genes as follows: a first sequence of genes transmitted from parent *p*_*1*_ (those preceding the crossing point) and a second sequence of genes transmitted from parent *p*_*2*_ (those after the point of crossing).
- *Stage 6*: Mutation of some created children
  When generating new individuals, in certain new created children, some of their genes can be randomly subjected to a mutation. So, the mutation process is essential so as to diversify the research^33^.
  In this work, we have tested our developed genetic algorithm as shown in Table 5, by applying simple mutation for certain formed new offspring and without mutation operators.
  Different mutation operators exist in the literature^34^. We applied in this study a simple mutation for certain reproduced children based on probabilities *p*_*m*_ calculated as follows^33^:
  5 $${p}_{m}=1 - {\sigma }^{-\frac{1}{l}}$$
  where *l* and *σ* are the chromosome size and the selective pressure, respectively.
- *Stage 7*: Generation of the next population
  This step consists to the generation of the new population, by selecting the best element of the last one and by completing the population by all the newly reproduced children.
- *Stage 8*: Decoding and displaying the best obtained recipe
  The termination criteria used in this model consists in fixing from the start the total number of generations *T*. So, if the number of generations is not reached, the steps from 3 to 7 should be repeated. Otherwise the program is stopped, the best obtained recipe was decoded and displayed.

## Results and discussion

To evaluate the efficiency of the proposed genetic algorithm, a set of 15 target colors was used to reproduce them by dyeing bicomponent filaments (PET/PTT) with the proposed recipes of disperse dyes. The CIELab coordinates of these reference colors are shown in Table 6. For each standard color, the GA should find the best recipe to dye bicomponent filaments (PET/PTT) that minimizes the $\Delta {E}_{CMC\left(2:1\right)}$ value between the predicted color and the standard sample.

**Table 6:** CIELab coordinates, *∑*(*K/S*) values and visual images of the used target color samples.

Target color n°	CIELab coordinates (D65/10°)					*∑(K/S)*^*(a)*^	Visual images
	L*	a*	b*	C*	*h*		
1	24.88	0.69	1.74	1.87	68.51	283.11	
2	44.61	53.63	1.52	53.65	1.63	102.31	
3	55.98	43.45	48.78	65.33	48.31	105.00	
4	47.72	43.88	36.28	56.94	39.58	129.95	
5	18.85	− 2.59	− 1.72	3.11	213.60	485.35	
6	42.33	46.23	29.29	54.72	32.36	166.77	
7	36.99	45.04	22.06	50.16	26.10	212.07	
8	41.68	− 20.07	15.34	25.26	142.61	120.99	
9	45.37	53.13	8.70	53.83	9.30	102.49	
10	78.96	9.88	101.44	101.92	84.44	200.60	
11	33.29	45.72	11.64	47.17	14.29	252.09	
12	32.83	4.88	1.74	5.18	19.65	163.62	
13	35.75	9.86	15.36	18.25	57.29	170.64	
14	27.47	15.57	− 28.89	32.82	298.31	204.70	
15	26.98	6.00	− 11.90	13.33	296.76	219.47	

^(a)^ The ***∑(K/S)*** value was calculated by summation of the *(K/S)* values at 10 nm intervals from the wavelength of 400 to 700 nm.

**Figure 5:**
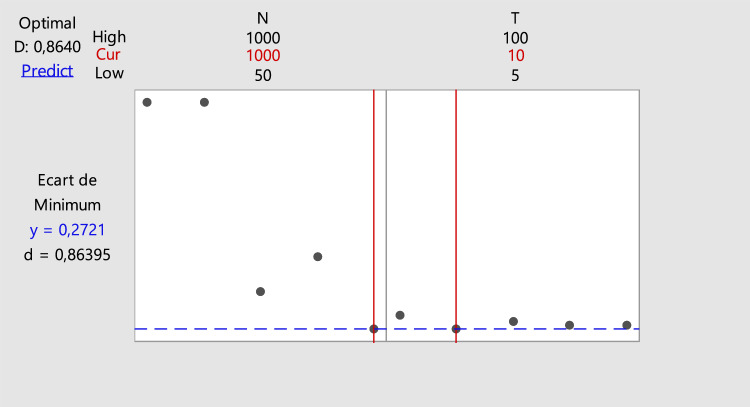
Optimization plot of genetic algorithm parameters (D: composite desirability; d: individual desirability; cur: current factor level settings; and y: minimal value of response).

The genetic algorithm parameters were set based on the results of the experimental design presented in Table 4. Figure 5 presents the optimization plot of the algorithm parameters using the Minitab Response Optimizer Tool. The optimization plot shows that *N* (number of individuals in each population) and *T* (total number of generations) should be fixed at 1000 and 10, respectively.

The obtained results by applying different selection and mutation techniques are shown in Table 7. Concerning the selection methods, we have similar results. So, choosing one of them will not affect the obtained results. The roulette wheel selection technique presents slightly better results since the CMC color differences do not exceed 0.93 contrary to rank and uniform selection methods where the maximum CMC color differences are 0.96 and 0.98, respectively. For the mutation, applying genetic algorithm with simple mutation allows to have better results; all the CMC color differences are smaller than 1. So, in this work we have opted for roulette wheel selection method and in order to maintain diversity within the population and prevent premature convergence, a simple mutation was made.

**Table 7:** Evaluation of the different GA operators using Δ*E*_*CMC(2:1)*_ color differences.

Target color n°	GA operators				
	Technique of selection			Technique of mutation	
	Roulette wheel selection	Rank selection	Uniform selection	Without mutation	Simple mutation
1	0.73	0.56	0.65	0.75	0.73
2	0.15	0.50	0.74	1.11	0.15
3	0.35	0.35	0.37	0.36	0.35
4	0.09	0.10	0.17	0.19	0.09
5	0.21	0.21	0.25	0.41	0.21
6	0.06	0.08	0.10	0.14	0.05
7	0.14	0.14	0.17	0.19	0.14
8	0.36	0.34	0.41	0.61	0.43
9	0.35	0.35	0.36	0.46	0.35
10	0.02	0.02	0.02	0.02	0.02
11	0.15	0.15	0.15	0.41	0.15
12	0.93	0.96	0.98	0.97	0.96
13	0.41	0.44	0.46	0.46	0.43
14	0.53	0.54	0.52	0.58	0.52
15	0.42	0.31	0.34	0.46	0.53
Mean	0.33	0.34	0.38	0.47	0.34
Maximum	0.93	0.96	0.98	1.11	0.96
Minimum	0.02	0.02	0.02	0.02	0.02
Standard deviation	0.25	0.23	0.25	0.29	0.26

Results obtained by the developed genetic algorithm for the 15 reference shades are summarized in Table 8, including the proposed dyeing recipes, CIELab coordinates, *∑*(*K/S*) values and visual images of reproduced colors as well as the $\Delta {E}_{CMC\left(2:1\right)}$ values between the targets and the predicted shades. Five simulations were performed for each target color. It is observed that all the reproduced shades showed good conformity with the target shades as far as the color differences are concerned. All the CMC color difference values were smaller than textile threshold of 1 which is considered good for textile industry^34–36^.

**Table 8:** Disperse dye recipes proposed by the genetic algorithm for dyeing bicomponent filaments, CIELab coordinates (D65/10°), *∑*(*K/S*) values and color differences values between corresponding solutions and desired reference colors.

Target color n°	Proposed recipes^(a)^ (%)			Samples dyed with proposed recipes							Δ*E*_*CMC(2:1)*_
	*DR167.1*	*DY211*	*DB79.1*	L*	a*	b*	C*	*h*	*∑(K/S)*^(b)^	Images	
1	0.68	1.28	1.37	17.25	0.78	2.01	2.16	68.83	301.01		0.73
2	0.50	0.00	0.00	44.28	50.48	1.62	50.51	1.84	99.16		0.15
3	0.29	0.58	0.00	48.46	39.80	46.39	61.12	49.37	111.92		0.35
4	0.35	0.30	0.00	45.96	42.90	35.65	55.78	39.73	136.74		0.09
5	0.35	0.45	1.05	17.10	− 2.85	− 1.87	3.41	213.34	514.06		0.21
6	0.59	0.26	0.00	42.34	45.62	29.41	54.28	32.80	161.97		0.06
7	0.95	0.20	0.00	37.76	47.55	22.91	52.78	25.73	209.68		0.14
8	0.00	0.36	0.28	33.78	− 20.42	16.34	26.15	141.32	135.74		0.36
9	0.65	0.05	0.00	41.45	48.69	11.25	49.97	13.02	96.99		0.35
10	0.00	2.00	0.00	78.85	10.47	101.57	102.1	84.11	200.13		0.02
11	2.50	0.00	0.00	30.49	46.63	11.49	48.03	13.85	250.05		0.15
12	1.48	0.64	0.69	14.20	5.14	1.84	5.46	19.75	150.91		0.93
13	0.44	0.48	0.13	31.42	9.68	14.18	17.17	55.69	199.96		0.41
14	0.33	0.00	0.28	27.31	11.39	− 22.21	24.96	297.14	200.02		0.53
15	0.50	0.08	0.45	28.99	6.90	− 13.61	15.26	296.89	220.11		0.42
Mean											0.33
Maximum											0.93
Minimum											0.02

^(a)^Dyes used in mixture are: *DR167.1* (C.I. Disperse Red 167.1), *DY211* (C.I. Disperse Yellow 211) and *DB79.1* (C.I. Disperse Blue 79.1).

^(b)^The ***∑(K/S)*** value was calculated by summation of the *(K/S)* values at 10 nm intervals from the wavelength of 400 to 700 nm.

These low values of CMC color differences (mean value = 0.33 and maximum value = 0.93) prove that the reproduced colors are conform to the standard colors. It can be therefore concluded that all the predicted disperse dye concentrations allow to obtain a good colorimetric correspondence of dyed bicomponent (PET/PTT) filaments with the standards.

## Conclusion

This work investigates the possibility of predicting bicomponent (PET/PTT) filaments dyeing recipes using genetic algorithm. The bicomponent polyesters filaments used in this study present high performances in terms of mechanical and thermal comfort properties. Their excellent values of elasticity and elastic recovery make them suitable for use as technical fabrics.

In order to predict the dyeing recipes allowing good matching with the desired target colors, a genetic algorithm has been developed and applied. The obtained results were promising. Indeed, the application of the developed genetic algorithm to reproduce 15 target colors showed very satisfactory results. All the predicted solutions presented low color differences values with the standard samples; all the $\Delta {E}_{CMC\left(2:1\right)}$ values are less than the unit, which is considered very acceptable for textile colors, with an average and maximum values of 0.33 and 0.93, respectively. These results prove that the developed genetic algorithm is an efficient technique for predicting color dyeing recipes of bicomponent (PET/PTT) filaments using disperse dyes in order to obtain a good conformity with the desired color target.

Additional work will be needed in order to generalize further points. Firstly, increasing the number of dyes may potentially introduce compatibility challenges in terms of dyeing kinetics and isotherms. Secondly, the accuracy of the color matching process heavily relies on the quality and quantity of available data. If the dataset used is limited or not representative of real-world conditions, the performance of the proposed model may be compromised. Lastly, transitioning the developed system into real-world industrial applications could face additional challenges such as scalability, integration with existing processes, and cost-effectiveness.

## Data Availability

All data generated or analyzed during this study are included in this published article.
